# Research progress on hyperbaric oxygen therapy for refractory peptic ulcer disease

**DOI:** 10.3389/fmed.2025.1708973

**Published:** 2025-11-21

**Authors:** Ze-Ming Chen, Wei-Feng Li, Xi-Qiu Yu, Xiao Tang, Qin-Hua Yang, Xiao-Lan Wei

**Affiliations:** Department of Gastroenterology, Shenzhen Luohu People's Hospital, Shenzhen, Guangdong, China

**Keywords:** hyperbaric oxygen therapy (HBOT), refractory peptic ulcer disease (RPUD), *Helicobacter pylori*, mechanism, review

## Abstract

Refractory peptic ulcer disease (RPUD) refers to gastric and duodenal ulcers that remain unhealed after 8–12 weeks drug treatment or are associated with complications despite drug treatment. Peptic ulcer disease is non-responsive to many treatments, has high recurrence rates, and often results in long-term pain, thus posing a persistent clinical challenge. Although the application of proton pump inhibitors has improved the healing rate and prognosis of patients with RPUD, drug efficacy is often limited by the emergence of drug resistant *Helicobacter pylori* and may be further reduced after co-application of non-steroidal anti-inflammatory drugs. Recent studies have demonstrated promising therapeutic outcomes of hyperbaric oxygen therapy (HBOT) in the management of refractory inflammatory bowel disease (IBD). Notably, emerging evidence further highlights the significant efficacy of HBOT in treating refractory peptic ulcers. In light of these findings, this review provides a systematic overview of the current research progress on HBOT for the treatment of RPUD.

## 1 Introduction

Peptic ulcer disease (PUD) is a common and painful gastrointestinal condition. The annual incidence of physician- diagnosed peptic ulcer disease is between 0.14 and 0.19% (1). There has been a steady decline in the incidence and prevalence of peptic ulcer disease (1). However, in the Chinese population, the popularization of digestive endoscopy and improved awareness of physical check-ups has resulted in increased PUD detection rates, with a PUD diagnosis for 10.3%−32.6% of people receiving gastroscopy examination (1). People who have peptic ulcer have upper abdominal pain, which is sometimes accompanied by dyspepsia (that is fullness, bloating, loss of appetite after eating a small amount of food, or nausea). The most serious complications of peptic ulcers are bleeding from the ulcer and perforation of the peptic ulcer (2). Thus, it is important to continue to monitor PUD and investigate effective treatment methods. With the application of proton pump inhibitors (PPIs), the treatment of PUD has improved greatly. However, not all PUD is responsive to PPIs. Gastric and duodenal ulcers that remain unhealed after 8–12-week drug treatment and those with complications even after treatment are characterized as refractory PUD (RPUD) (2). *H pylori* is the primary cause of PUD (3). Currently, bismuth-containing quadruple therapy (PPI + bismuth + 2 antibiotics) is recommended as the standard therapy for *H. pylori* eradication (4). The curative rate of RPUD is low mainly because of the persistent infection caused by *Helicobacter pylori* during the ulcer healing process. Antimicrobial resistance is a leading cause of treatment failure (5). Therefore, improved strategies for RPUD treatment are critically needed. Recently, hyperbaric oxygen therapy (HBOT) and has been shown to promote the healing of RPUD when used in combination with standard RPUD therapy. Chinese researchers have been conducting clinical trials on the efficacy of combining HBOT in PUD treatment since 1982; nevertheless, new therapeutic mechanisms of HBOT in combination with standard RPUD treatments continue to emerge. Systematic and comprehensive research on the healing of RPUD through HBOT combination therapy may help to identify a strategy that avoids drug resistance of *H. pylori*, thus providing a potentially promising approach for improving therapeutic outcomes in the future.

## 2 Role of HBOT in RPUD treatment

### 2.1 The etiology of RPUD

The etiology and pathogenesis of RPUD may include the following features: (1) *Risk factors for RPUD and treatment compliance in the patients*. The risk factors for RPUD include smoking, alcohol, and psychological stress. Influencing factors for treatment compliance also include education level, place of residence, medical payments, doctor-patient relationship, and knowledge of the disease. (2) *Persistent H. pylori infection*. Persistent *H. pylori infection* is the main cause of peptic ulcers (3). Approximately 35%−70% of the global population is infected by *H. pylori* (6). Furthermore, antimicrobial resistance is the main cause of persistent *H. pylori* infection (7). (3) *Non-H. pylori-related factors*. A review of the medical literature indicates that up to 52% of duodenal ulcers and 47% of gastric ulcers are not caused by *H. pylori* infection. While a variety of alternate contributing factors have been proposed, the consumption of non-steroidal anti-inflammatory drugs (NSAIDs), such as aspirin, is the most common cause of *H. pylori*-negative duodenal ulcer (8). Another study comparing patients with and without NSAID use [odds ratio (OR) = 0.74, 95% confidence interval (CI): 0.46–1.20] indicated that 6.4%−11.8% of patients using NSAIDs developed PUD, which is especially the case for NSAID-naïve patients (OR = 0.26, 95% CI: 0.14–0.49) (9). Other causes include false-negative *H. pylori* infection, genetic susceptibility, and rare idiopathic gastric acid hypersecretion or gastrinoma.

### 2.2 The healing process of PUD and the pathological causes for healing challenge in RPUD

Once an ulcer develops, its repair and healing are governed by a coordinated sequence of fundamental biological processes. Initially, through inflammatory chemotaxis, polymorphonuclear leukocytes and macrophages infiltrate the ulcer site, where they enzymatically degrade and remove necrotic debris from the ulcer base, facilitating the formation of granulation tissue. Subsequently, myofibroblasts migrate into the lesion, contributing to granulation tissue formation and synthesizing collagen fibers and other extracellular matrix components, ultimately leading to scar formation (10). Various pharmacological agents and plant extracts have been shown to attenuate the progression of gastric mucosal injury and accelerate healing by downregulating the secretion of pro-inflammatory cytokines such as IL-6 and TNF-α (11). Epidermal growth factor, an endogenous bioactive molecule, plays a critical role in gastric mucosal protection by inhibiting gastric acid secretion, stimulating cellular proliferation and tissue repair, and preserving mucosal integrity against damaging stimuli. Additionally, nitric oxide exerts protective effects on the gastric mucosa, primarily through the regulation of gastric microcirculation, thereby supporting angiogenesis, vascular remodeling, and mucosal regeneration during the healing process (11).

Any factor that inhibits the cell regeneration process may directly affect the ulcer healing process. For example, inflammatory stimuli often cause proliferative endarteritis, thickening of small arterial walls, narrowing of the lumen, or thrombosis, resulting in insufficient local blood supply, affecting tissue regeneration, and challenging ulcer healing. Additionally, *H. pylori* possesses pathogenic genes—vacuum cytotoxin-associated protein A (*VacA*) and cytotoxin-associated protein A (*CagA*)—that are responsible for epithelial cell injury and chronic inflammation during *H. pylori* infection. These genes are important determinants of *H. pylori* toxicity and challenge ulcer healing, possible leading to cancer (6). Any changes in invasive factors (gastric acid and pepsin) and protective factors (mucus and bicarbonate) in the cavity may also delay ulcer healing. Finally, COX derived prostaglandin inhibition, vascular damage, and topical effects are the main players in the pathogenesis of ulcers caused by NSAIDs (12).

### 2.3 Emergence of HBOT as a novel therapeutic enhancer

**Figure 1:**
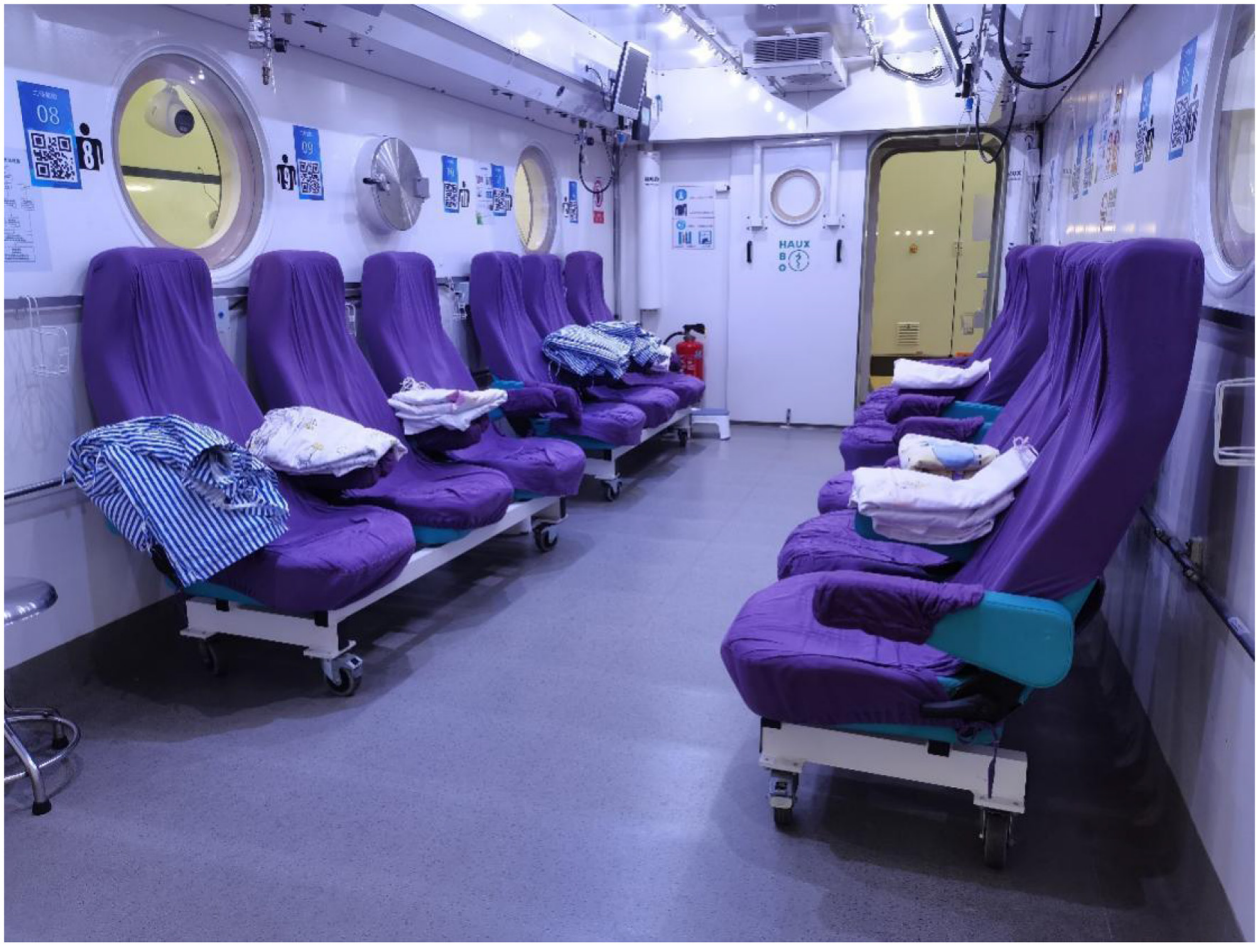
Multiplace chamber.

**Figure 2:**
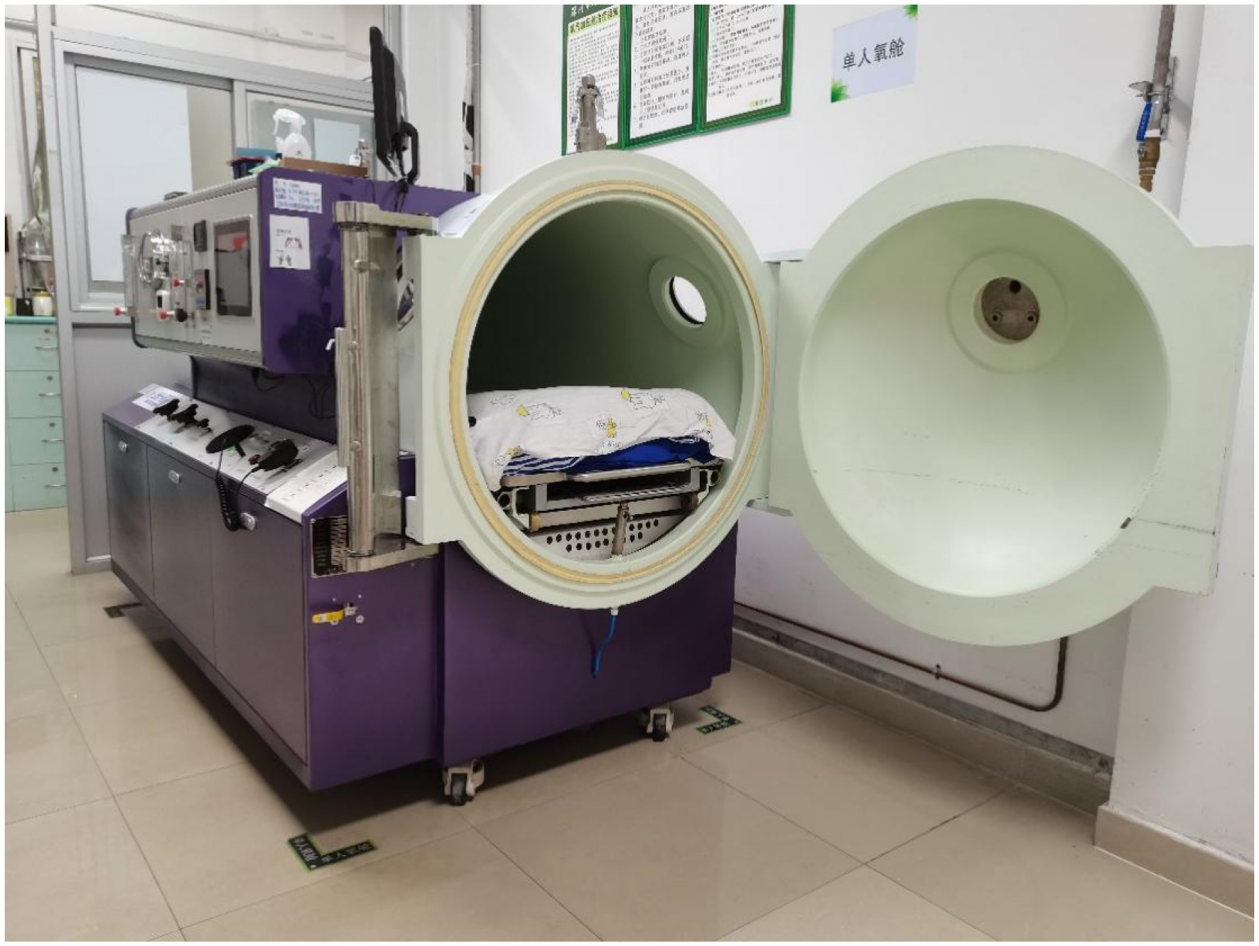
Monoplace chamber.

HBOT refers to a treatment in which patients inhale 100% oxygen in a hyperbaric oxygen chamber that is pressurized above sea level (one atmosphere absolute, ATA) (13). To exert clinically therapeutic efficacy, the Undersea and Hyperbaric Medical Society stipulates that the pressure must be ≥1.4 ATA, and the applied pressure is usually in the range of 2–3 ATA in clinical practice (14). Treatment is delivered in multiplace chambers (Figure 1) or in monoplace chambers (Figure 2). In a monoplace chamber, a single patient breathes compressed pure oxygen. In a multiplace chamber, multiple patients are exposed to pressurized air together while they each breathe pure oxygen through a face mask, hood, or endotracheal tube. HBOT has been applied to the treatment of many diseases with positive effects. In 2017, the tenth European Consensus Conference on Hyperbaric Medicine revised the acceptable, unacceptable, and non-recommended conditions of HBOT (15), including carbon monoxide poisoning, decompression sickness, diabetic foot pathology, and open fractures with crush injuries; however, RPUD has not been included in either the acceptable or non-recommended lists for HBOT. In the following sections, we compile evidence suggesting that HBOT may also have a significant effect on the healing of PUD, and especially RPUD.

### 2.4 HBOT efficacy for PUD treatment

Oxygen is required for cell growth, proliferation, and differentiation. Consequently, HBOT improves the microenvironment of healing and growth at various layers of peptic ulcers and plays an important role in reducing the occurrence and progression of refractory peptic ulcer healing. In 1982, Jin et al. used hyperbaric oxygen to treat 100 patients with PUD in a comparative analysis of the short term and long-term effect of HBOT. The results showed that combining HBOT with the conventional treatment promoted the healing of peptic ulcers and reduced the recurrence rate of PUD. Although the latter study was the first to report a close relationship between HBOT and PUD in a Chinese cohort, the therapeutic efficacy of PUD drugs has been greatly improved by the extensive application of PPIs. In 2006, Huang et al. demonstrated that combined HBOT and PPI therapy significantly promoted the healing of RPUD and enhanced the eradication rate of *H. pylori*. The aforementioned studies were conducted in China. Additionally, there are reports from other countries on the use of hyperbaric oxygen therapy for the treatment of peptic ulcers. Elizavetina et al. indicated that HBOT and pyridinolcarbamate were effective in the treatment of elderly patients with peptic ulcer complicated by coronary heart disease and atherosclerosis (16). Several studies have also reported that HBOT and nootropic agents are effective in the treatment of peptic ulcers (17). Notably, a study analyzing the treatment outcomes of 179 patients with peptic ulcer bleeding demonstrated that the use of hyperbaric oxygen therapy in managing ulcer bleeding reduced the post-operative complication rate by 3.3-fold and significantly decreased mortality (18). Since then, HBOT in PUD treatment, especially in RPUD, has slowly been integrated in clinical studies.

### 2.5 HBOT mechanisms in treating RPUD

**Figure 3:**
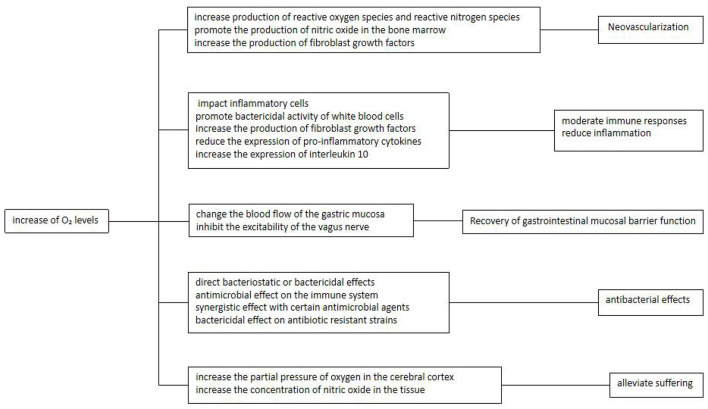
HBOT mechanisms in treating RPUD.

During ulcer repair and healing, the demand and utilization of oxygen increase. According to Henry's law of respiration, oxygen is not stored in the tissue. In HBOT, the partial pressure of inhaled oxygen is increased through supplementation of 100% oxygen under pressure. Thus, regular HBOT provides a means to continuously provide oxygen to the ulcer site through the circulation in order to promote ulcer healing (13). Emerging studies have suggested that the beneficial effects of oxygen at the site of an ulcer, as increased by HBOT, are mediated by a variety of potential mechanisms that are described in detail in Sections 2.5.1 to 2.5.5 below (Figure 3).

#### 2.5.1 HBOT promotes angiogenesis

HBOT stimulates peptic ulcer healing by promoting angiogenesis, which may be attributed to five underlying mechanisms:

First, formation of granulation tissue is essential for ulcer healing; however, neovascularization requires the migration and proliferation of dermal and epidermal cells to provide oxygen and nutrients. Thus, HBOT promotes the transformation of ulcers to the proliferative stage by promoting expansion of dermal and epidermal cells.

Second, increases in oxygen concentration lead to increased production of reactive oxygen species (ROS) and reactive nitrogen species, both of which play an essential role in promoting the formation of new blood vessels and matrix (13).

Third, hypoxia is a stimulating factor for angiogenesis. HBOT creates a gradient between the hypoxic tissue at the center and the surround tissues with hyperoxia, thereby becoming a driving force for angiogenesis (13).

Fourth, increase in oxygen promotes the production of nitric oxide in the bone marrow, thereby stimulating the mobilization of vascular-derived stem cells/progenitor cells. Thus, more stem cells are recruited to the ulcer to accelerate the formation of blood vessels (19, 20).

Fifth, hyperbaric oxygen increases the production of fibroblast growth factors to promote the migration and proliferation of fibroblasts and increase the rate of collagen production, thereby increasing the tensile strength of ulcers (13).

#### 2.5.2 HBOT moderates immune responses and reduces inflammation

*H. pylori* recruits immune cells to the gastric mucosa initiate immune responses and promote gastric inflammation. However, hyperoxia promotes decreased inflammation by impacting the three main inflammatory cells (macrophages, white blood cells and neutrophils) and by inducing vasoconstriction to decrease local edema (21). In addition, increased oxygen enhances to reduce the chemotaxis and adhesion of leukocytes, thus alleviating ischemia-reperfusion injury and suppressing the formation of inflammatory mediators (13).

HBOT also reduces inflammatory tissue injury that is associated with infection by inducing the overexpression of growth factors while downregulating immune cytokines, thus modulating the immune response. The increase in oxygen levels during HBOT has been shown to modulate key immune parameters, including the suppression of interferon-γ and pro-inflammatory cytokines such as IL-1, IL-6, and TNF-α, a transient decrease in the CD4:CD8 T cell ratio, reduced levels of serum soluble IL-2 receptor, enhanced plasma fibronectin concentration, and a significant upregulation of anti-inflammatory IL-10 (22). Whereas, HBOT reduces the expression of pro-inflammatory cytokines, it increases the expression of interleukin 10, which has been confirmed in animal models of septic shock and ischemic injury (23). The oxygen level of the environment is a key factor in the antibacterial activity of neutrophils. Sterilization promotes a potent respiratory burst by generating superoxide free radicals that require a large amount of oxygen. Studies have also demonstrated that the bactericidal ability of neutrophils is significantly improved after oxygen levels are increased by HBOT application; HBOT promotes apoptosis of human macrophages and induces and accelerates the death of lymphocytes via the mitochondrial pathway. These findings indicate that HBOT exerts an immunomodulatory effect (24).

#### 2.5.3 HBOT inhibits the secretion of gastric acid and pepsin, with potential effect on vagus nerve function, thus facilitating the recovery of gastrointestinal mucosal barrier function

*H. pylori* releases of platelet-activating factors that promote thrombosis in surface capillaries, resulting in vascular obstruction, mucosal ischemia, and other injury to the gastric mucosal barrier. Long-term usage of NSAIDs such as aspirin, which also has been identified as a root cause for ulcers, not only directly stimulates the gastric mucosa, but also inhibits the synthesis of gastric mucosal prostaglandin and affects blood circulation. As an additional risk factor, smoking, may also reduce mucosal blood circulation and weaken the mucosal defense barriers. Thus, restoration of mucosal barrier is an important focus in the treatment of RPUD. Evidence suggests that HBOT may have positive effects on the mucosal barrier. In a rat gastric-acid secretion model, exposure to hyperbaric oxygen significantly reduced the gastric digestive activity in the rats, supporting the idea that hyperbaric oxygen may change the blood flow of the gastric mucosa (25).

As an additional consideration, excessive gastric acid secretion, which may be caused by hypertension or anxiety, gastric juice secretion disorders, or vagus nerve dysfunction, often occur in patients with ulcer disease. Mental stimulation causes cerebral cortex dysfunction, leading to autonomic nervous dysfunction. Hyperfunction of the vagus nerve promotes an increase of gastric acid secretion, which is associated with the occurrence of duodenal ulcers; on the other hand, reduced excitability of the vagus nerve and weakened gastric motility increase the secretion of gastrin, thereby promoting an increase in gastric acid secretion and the formation of gastric ulcers. Gastric mucosal parietal cells, which have enzyme inhibition properties, may also inhibit pepsin secretion (26). Evidence suggests that HBOT may reverse many of these effects on vagus nerve function to assist in restoring the gastric mucosal barrier. Hyperbaric oxygen may regulate the activity of the cerebral cortex and the autonomic nervous system, thereby inhibiting the excitability of the vagus nerve, reducing gastric acid secretion, and preventing smooth muscle spasms, which would be conducive to ulcer healing.

#### 2.5.4 HBOT exerts antibacterial effects

The antibacterial ability of HBOT provides an additional activity that may impact its efficacy in treating RPUD and is attributed to four related mechanisms. First, HBOT exerts direct bacteriostatic or bactericidal effects on anaerobic, and some aerobic bacteria, including. *H. pylori*, which is a microaerophile (27). The direct antibacterial effect of HBOT is thought to be a result of ROS formation. HBOT induces oxidative stress by disturbing the balance between the formation and degradation of ROS, thus increasing the levels of cellular ROS (28). Consequently, HBOT is effective in bacteria lacking antioxidant defense pathways (29) and induces antibacterial activity in a dose-dependent manner (30, 31). The cellular targets of the ROS toxic effects are deoxyribonucleic acid, ribonucleic acid, protein, and lipids (32), with deoxyribonucleic acid as the main target of hydrogen peroxide-dependent cytotoxicity. This cytotoxicity destroys the structure and base of deoxyribose; active oxygen induces physical injury to free nucleotides and also destroys single-stranded or double-stranded DNA in the double helix via the by-products of lipid peroxidation induced by ROS (33). High concentrations of ROS also directly injure lipid: destructive hydroxyl groups trigger lipid peroxidation and stimulate the oxidation of polyunsaturated phospholipids in the cell membrane, leading to functional failure (34). Reactive oxygen destroys the lipid bilayer structure of the cell membrane and makes the receptors and proteins located on the membrane ineffective, ultimately leading to cell fluidity, cytoplasmic content outflow, and loss of enzyme function (35, 36). Proteins are also molecular targets of ROS, which cause injury to amino acid residues (37). Thus, HBOT mediates direct antibacterial effects by promoting oxidative changes to several target molecules related to bacterial function.

Second, HBOT exerts an antimicrobial effect on the immune system, as discussed in Section 2.5.2 above. This involves modulation of macrophages, white blood cells and neutrophils and results in increased immune-mediated reduction in *H. pylori* level.

Third, HBOT has a synergistic effect with certain antimicrobial agents. In clinical practice, HBOT is usually used in combination with antibiotic therapy for infections. Thus, induction of hyperoxia during HBOT may affect the activity of antibiotics (38). Another study demonstrated that for some bactericides, such as β-lactams, quinolones, and aminoglycosides, the bactericidal activity relies, in part, on the aerobic metabolism of the bacteria. Therefore, the efficiency of these drugs is affected by both the presence of oxygen and the metabolic characteristics of the pathogens (39). Consequently, the potential *in vivo* oxygen concentration in the infected tissue and the impact on pathogen antibiotic sensitivity are key factors in determining antimicrobial treatment characteristics. HBOT induces aerobic metabolism of bacteria to reoxygenate hypoxic infected cells, which may explain its synergistic effect with antimicrobial agents.

Fourth, HBOT has shown a bactericidal effect on antibiotic resistant strains. Due to the development and spread of antibiotic-resistant bacterial pathogens, antimicrobial drugs often lose their effect over time (40). HBOT may be suitable for the treatment and prevention of multidrug resistant pathogens in the case of antibiotic treatment failure (41). In fact, a previous study has shown that HBOT has bactericidal effects on clinically important drug-resistant bacteria, such as methicillin-resistant *Staphylococcus aureus*, which are significantly reduced after the exposure to HBOT (90 min under two oxygen tensions) (42).

#### 2.5.5 HPOT inhibits ulcer-related nociceptive pain, thereby providing a mechanism for alleviating patient suffering

HBOT has been shown to inhibit nociceptive pain in mouse models. It increases the partial pressure of oxygen in the cerebral cortex and the concentration of nitric oxide in the tissue (43) and exerts an anti-nociceptive effect mediated by neuronal nitric oxide-dependent release of opioid peptides (44). The role of nitric oxide in HBOT-induced analgesia was confirmed in a study in which the analgesic effect induced by HBOT was attenuated by preconditioning of the lateral ventricle and intrathecal and targeting of neuronal nitric oxide synthase with an antisense nucleotide (45). These results indicate that HBOT exerts a variety of mechanisms to inhibit nociceptive pain and alleviate symptoms of discomfort (43), which could also have relevance to the treatment of pain symptoms in RPUD patients.

**Figure 4:**
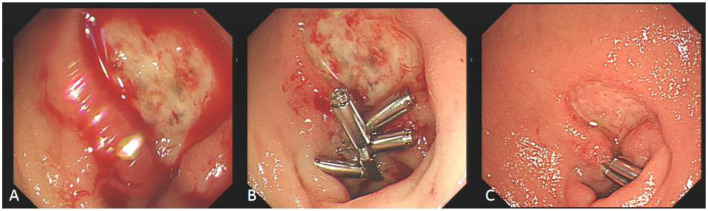
Comparison before and after hyperbaric oxygen treatment of duodenal bulb ulcer bleeding. **(A)** This is a large, intractable ulcer with hemorrhage in the duodenal bulb. **(B)** The bleeding stopped temporarily after clamping with some titanium clamps. **(C)** After 2 week of hyperbaric oxygen treatment, duodenal bulb ulcer improved significantly.

**Figure 5:**
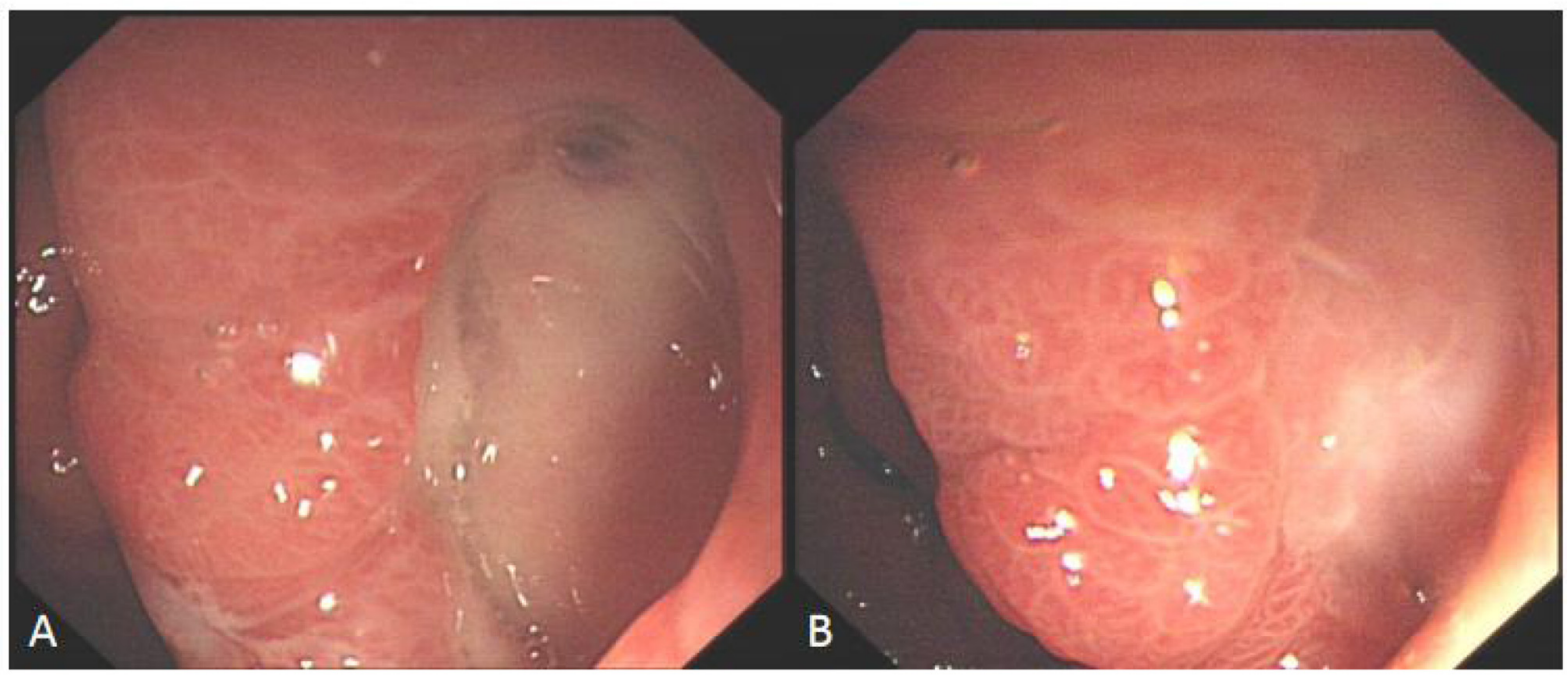
Comparison before and after hyperbaric oxygen treatment of ulcer on the posterior wall of gastric antrum. **(A)** Intractable ulceration of the posterior wall of the gastric antrum, approximately 1.8 cm × 2.5 cm, the broken ends of blood vessels are visible on the surface. **(B)** After 2 weeks of hyperbaric oxygen treatment, the ulcer was about 6 mm × 12 mm in size, with granulation tissue growing on the surface and no broken ends of blood vessels.

**Figure 6:**
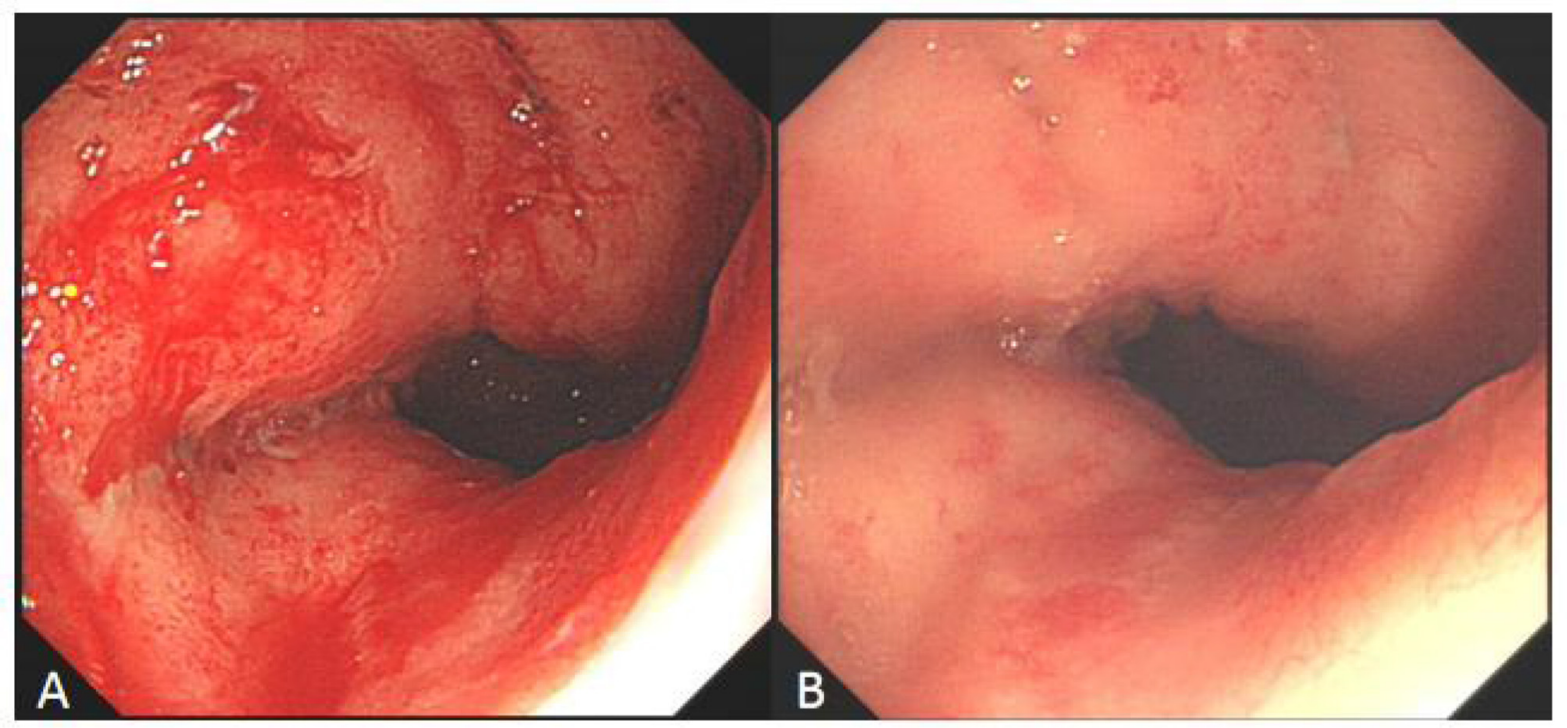
Comparison of refractory ulcer after radiotherapy at esophagogastric anastomosis before and after treatment. **(A)** Esophagogastric anastomosis showed intractable ulceration and bleeding after radiotherapy. **(B)** After 2 weeks of hyperbaric oxygen treatment, the ulcer lesions healed markedly and the bleeding stopped.

Some cases of hyperbaric oxygen therapy for refractory peptic ulcer disease (Figures 4–6).

## 3 Prospect

In recent years, HBOT has been increasingly applied in the treatment of various conditions, such as refractory wounds, radiation-induced tissue damage, gas gangrene, and carbon monoxide poisoning, leading to growing clinical and scientific interest. In gastrointestinal disorders, HBOT is emerging as a promising adjuvant therapy for inflammatory bowel disease (IBD). Evidence indicates that HBOT reduces levels of pro-inflammatory cytokines and markers of oxidative stress in IBD patients, contributing to disease improvement (46). Furthermore, studies demonstrate that HBOT can ameliorate gut microbiota dysbiosis in Crohn's disease (CD), alleviating both intestinal and systemic inflammation. HBOT is well-tolerated in CD patients and exhibits a favorable safety profile, with potential synergistic effects when combined with ustekinumab (47). A clinical study involving patients with long-standing CD complicated by anal fistula (disease duration ≥3 years) reported that adjunctive HBOT significantly improved clinical symptoms compared to conventional therapy alone (48) Moreover, a meta-analysis revealed high clinical remission rates following HBOT in ulcerative colitis (UC) and CD, ranging from 87 to 88%. Although the remission rate for CD-related anal fistula was lower, it remained clinically meaningful at 48%−60% (49, 50). Given that IBD is primarily characterized by mucosal ulceration, and considering the demonstrated efficacy of HBOT in promoting healing of intestinal ulcers, it is reasonable to hypothesize that HBOT may also be beneficial in the management of refractory gastric and duodenal ulcers.

Existing research has advanced our understanding of the mechanisms underlying HBOT in the treatment of RPUD; however, knowledge regarding its therapeutic benefits remains limited and incomplete. Persistent *H. pylori* infection is a primary driver of ulcer recurrence and impaired healing in refractory cases. Moreover, antibiotic resistance significantly hinders the healing process and is strongly associated with high relapse rates. Numerous clinical studies have demonstrated that adjunctive HBOT in the management of refractory peptic ulcers not only accelerates ulcer healing but also reduces recurrence and alleviates patient symptoms, offering a promising new therapeutic strategy. These findings underscore the importance of further investigating the combination of HBOT with conventional treatments to enhance healing outcomes and potentially mitigate the development of antibiotic resistance. Nevertheless, the precise therapeutic mechanisms of HBOT are not yet fully understood and require validation through rigorous clinical research. For instance, the biological processes involved in the transition from ulcer repair to scar maturation remain poorly characterized. Metronidazole is widely used to treat anaerobic bacterial and protozoal infections due to the antimicrobial activity of its reduced metabolites in hypoxic environments (51). While preclinical *in vitro* and *in vivo*s studies suggest that combining HBOT with metronidazole may alleviate peptic ulcer-related pain, large-scale randomized controlled trials are still lacking. Furthermore, the indications and contraindications for HBOT in the context of peptic ulcer disease have not been systematically established. Current research on HBOT for refractory peptic ulcers remains limited. A substantial number of well-designed randomized controlled trials are still required to determine the optimal treatment protocols and assess the safety profile of HBOT, in order to facilitate the standardization of its clinical application. We have reason to believe that HBOT combined with the standard treatment of RPUD is likely to improve curative rates, reduce recurrence rates, and alleviate the suffering of patients with RPUD, thus supporting a new direction of clinical application in the future. Given the current knowledge, HBOT combined with standard acid-suppression regimens may become recognized as a viable cure for RPUD.
